# Edu-couch-ing the masses: an online, multi-disciplinary psychiatry teaching programme

**DOI:** 10.1192/bjo.2021.369

**Published:** 2021-06-18

**Authors:** Chiara Cattra, Laur Evans

**Affiliations:** 1 Solent NHS Trust, Sussex Partnership NHS Foundation Trust; 2 South West London and St George's Mental Health NHS Trust

## Abstract

**Aims:**

In response to medical students expressing concer at limited access to psychiatric placements, particularly on-the-ground teaching or witnessed patient cases, we established “Psych From The Couch” – an open-access, free, Zoom-based, interactive teaching programme. We sought to:

Explore new means of psychiatric education, assess needs of multiple “categories” of student – medical, nursing, or PA students, junior doctors, wider MDT – and meet those needs in a creative, yet virtually-limited format.

Assess disparities between students' self-declared learning deficits and objective knowledge gaps.

To explore the use and value of virtual programmes as a structured means for inclusive multi-disciplinary education of psychiatric practice.

**Method:**

We gathered information on students' self-declared learning needs and deficits, location, role, training level, and confidence at the outset of the programme, with data from ~180 “students”.

We experiemtned with learning styles and methods of online interaction, running a series of 10 sessions - recorded for those unable to attend - incorporating the bredth of psychiatric curricula:

Diagnostic Principles

“Organic” Psychiatry

Substance Misuse

Psychotic Disorders

Affective Disorders

Old Age Psychiatry

CAMHS

Emergencies & Legalities

Examinations in Psychiatry

Real World Psychiatry

We utilised initial sign-up forms and repeated feedback requests to assess wider student needs, establish overarching structure to our programme, and ensure learning objectives were appropriate and met.

We collated final feedback and scores at the close, assessing via examination questions and self-defined Likert scale, and incentivising feedback with a final portfolio certificate.

**Result:**

Demographics of open-access teaching varied broadly, from senior medical staff to access to medicine students; 92.9% were medical students. Students were diversely sourced from all years', with ~50% collectively in their penultimate or final years' of study.

Most common self-defined decficits reported were understandably anxiety regarding practical examinations or assessment given recent placement restrictions, however many reflected on anxieties regarding psychiatric emergencies, substance misuse, legal frameworks, personality disorders as a diagnostic category, and pharmacological management.

Our cohort responded warmly to our teaching style and techniques, with feedback and consequent improvements to teaching technique weekly. We were able to evidence improvements to global confidence, and confidence in key areas of prior learning anxiety.

**Conclusion:**

Categorising self-defined deficits yielded fasctinating information on students' perception of their learning needs and deficits; these data may offer insight into potential deficits in the scope of nationwide psychiatric teaching.

We were able to separately identify international students' or professionals' self-defined needs as distinct from UK students and graduates, with further rich data on the potential needs of those entering the NHS workforce.

We also evidenced – with data regarding increased confidence, fewer self-defined learning deficits, significant Twitter social interaction, and in practical application of a virtual teaching methodology – proof of the concept of “Psych From The Couch”.

